# Supplementary material for: The burden of mental disorders in Nepal between 1990 and 2019: Findings from the Global Burden of Disease Study 2019
Source: Glob Ment Health (Camb). 2023 Sep 12;10:e61. doi: 10.1017/gmh.2023.55 (PMC10579670; doi:10.1017/gmh.2023.55)
Supplement: Dhungana et al. supplementary material 1 — Dhungana et al. supplementary material [file S2054425123000559sup001.docx]

**Supplementary file 1. Prevalence of specific mental disorder in 2019**

| **Mental disorders** | **Both sex** | | **Male** | | **Female** | |
| --- | --- | --- | --- | --- | --- | --- |
|  | **All ages** | **Age standardized** | **All ages** | **Age standardized** | **All ages** | **Age standardized** |
| Major depressive  Disorders | 3430.14  (2956.18, 3984.23) | 3795.9  (3265.67, 4408.13) | 2630.43  (2252.22, 3074.06) | 3019.16  (2576.52, 3534.1) | 4158.48  (3571.36, 4844.39) | 4465.38  (3835.6, 5198.82) |
| Anxiety disorders | 3176.53  (2520.42, 4034.94) | 3277.32  (2624.16, 4134.75) | 2557.78  (2000.03, 3198.12) | 2726.06  (2175.66, 3413.44) | 3740.06  (2940, 4846.47) | 3757.4  (2990.02, 4841.11) |
| Idiopathic  developmental intellectual  disability | 2605.51  (1705.16, 3516.27) | 2503.69  (1635.85, 3380.81) | 2845.31  (1858.98, 3858.42) | 2715.02  (1770.78, 3684.33) | 2387.12  (1564.88, 3210.37) | 2307.85  (1510.39, 3106.82) |
| Other mental  Disorders | 1304.19  (1008.28, 1672.93) | 1444.53  (1119.48, 1845.86) | 1499.12  (1137.12, 1928.18) | 1721.79  (1308.24, 2200.19) | 1126.66  (864.7, 1458.99) | 1210.48  (932.86, 1565.35) |
| Dysthymia | 1168.95  (901.04, 1500.48) | 1248.27  (979.36, 1585.81) | 944.52  (722.35, 1213.3) | 1050.31  (810.6, 1356.99) | 1373.34  (1064.74, 1782.68) | 1416.17  (1112.73, 1806.18) |
| Attention-deficit  hyperactivity disorder | 709.33  (495.22, 975.91) | 630.3  (441.44, 863.51) | 1050.45  (728.26, 1462.91) | 916.43  (641.66, 1261.22) | 398.66  (274.75, 543.05) | 361.06  (249.59, 492.77) |
| Conduct disorder | 574.07  (406.77, 769.73) | 483.57  (342.68, 642.9) | 804.53  (587.73, 1046.22) | 640.56  (467.78, 832.62) | 364.18  (232.86, 517.1) | 324.11  (208.71, 455.92) |
| Bipolar disorder | 372.6  (286.13, 476.05) | 383.91  (299.72, 482.41) | 378.04  (288.36, 482.12) | 401.44  (312.27, 504.18) | 367.66  (280.27, 470.87) | 369.24  (286.43, 465.39) |
| Autism spectrum disorders | 295.24  (240.51, 355.47) | 288.59  (235.02, 347.58) | 432.13  (352.63, 520.28) | 420.99  (344.3, 507.99) | 170.58  (135.98, 208.11) | 167.48  (133.61, 204.15) |
| Schizophrenia | 249.6  (197.37, 307.15) | 266.99  (212.2, 326.95) | 270.12  (213.78, 331.69) | 302.87  (240.88, 370.67) | 230.92  (181.45, 283.46) | 237.29  (188.78, 291.08) |
| Bulimia nervosa | 79.3  (52.96, 109.91) | 72.55  (48.08, 101.4) | 61.35  (39.34, 89.47) | 58.63  (37.71, 84.4) | 95.66  (63.86, 133.07) | 84.05  (56.52, 116.89) |
| Anorexia nervosa | 36.15  (24.69, 52.52) | 31.31  (21.5, 44.97) | 18.39  (12.51, 27.26) | 15.98  (11.01, 23.54) | 52.33  (35.42, 76.58) | 44.58  (30.33, 64.87) |

**Supplementary file 2. DALYs lost due to mental disorders between 1990 and 2019**

|  | **Both sex** | | | **Male** | | | **Female** | | |
| --- | --- | --- | --- | --- | --- | --- | --- | --- | --- |
| **Year** | **All ages** | **age standardized** | **% of total DALYS** | **All ages** | **age standardized** | **% of total DALYS** | **All ages** | **age standardized** | **% of total DALYS** |
| **1990** | 1421.66  (1036.74, 1870.04) | 1739.45  (1285.35, 2287.93) | 1.79  (1.34, 2.32) | 1375.63  (997.36, 1803.81) | 1665.07  (1232.96, 2171.55) | 1.7  (1.26, 2.21) | 1467.92  (1068.11, 1944.53) | 1814.42  (1329.34, 2396.07) | 1.89  (1.41, 2.44) |
| **1995** | 1414.42  (1036.32, 1861.19) | 1720.37  (1278.89, 2261.04) | 2.24  (1.65, 2.89) | 1370.19  (990.41, 1806.12) | 1649.38  (1211.89, 2173.98) | 2.12  (1.56, 2.74) | 1458.8  (1063.45, 1929.83) | 1791.28  (1320.04, 2350.7) | 2.38  (1.76, 3.04) |
| **2000** | 1502.69  (1103.09, 1980.14) | 1807.28  (1344.53, 2375.17) | 3.07  (2.32, 3.97) | 1430.49  (1039.03, 1871.39) | 1700.54  (1248.64, 2221.07) | 2.81  (2.1, 3.63) | 1575.08  (1158.01, 2083.93) | 1914.34  (1415, 2534.02) | 3.36  (2.55, 4.32) |
| **2005** | 1556.85  (1140.29, 2056.8) | 1816.41  (1342.39, 2391.03) | 3.9  (2.96, 4.92) | 1461.88  (1074.97, 1920.99) | 1694.14  (1239.53, 2217.52) | 3.47  (2.63, 4.44) | 1650.06  (1203.7, 2166.8) | 1935.43  (1417.36, 2542.37) | 4.36  (3.32, 5.48) |
| **2010** | 1636.35  (1203.26, 2147.28) | 1846.03  (1370.45, 2417.45) | 4.68  (3.62, 5.9) | 1512.26  (1115.56, 1995.31) | 1709.91  (1260.47, 2253.64) | 4.08  (3.13, 5.17) | 1754.37  (1288.29, 2298.86) | 1971.05  (1450.49, 2588.47) | 5.33  (4.1, 6.68) |
| **2015** | 1636.44  (1199.32, 2160.23) | 1766.57  (1310.42, 2337.09) | 4.83  (3.7, 6.1) | 1510.85  (1102.57, 2007.79) | 1652.74  (1208.28, 2177.99) | 4.12  (3.14, 5.25) | 1752.71  (1284.28, 2316.69) | 1864.48  (1373.33, 2471.36) | 5.61  (4.32, 7.09) |
| **2019** | 1691.08  (1244.47, 2224.51) | 1773.74  (1309.23, 2335.08) | 5.53  (4.22, 6.98) | 1545.37  (1129.01, 2027.12) | 1653.62  (1210.22, 2171.39) | 4.73  (3.58, 6) | 1823.78  (1330.85, 2413) | 1873.84  (1382.59, 2478.2) | 6.37  (4.89, 7.96) |

**Supplementary file 3. DALYs attributable to specific mental disorders (100,000 population)**

|  | **Both sex** | | **Male** | | **Female** | |
| --- | --- | --- | --- | --- | --- | --- |
| **Mental disorders** | **All ages** | **Age standardized** | **All ages** | **Age standardized** | **All ages** | **Age standardized** |
| Major depressive disorder | 687.86  (465.55, 953.1) | 754.62  (510.96, 1045.8) | 531.59  (350.9, 739.48) | 605.29  (403.62, 842.67) | 830.19  (560.27, 1159.32) | 883.21  (598.08, 1228.91) |
| Anxiety disorders | 302.23  (199.03, 429.32) | 309.51  (205.76, 434.63) | 245.44  (162.14, 345.61) | 259.69  (172.72, 364.95) | 353.95  (230.59, 507.35) | 352.97  (229.71, 498.14) |
| Idiopathic developmental  intellectual disability | 103.87  (57.01, 168.63) | 99.9  (54.97, 162.85) | 114.21  (62.07, 186.85) | 109.17  (59.37, 178.21) | 94.46  (52.36, 152.89) | 91.33  (50.67, 147.78) |
| Dysthymia | 112.57  (68.7, 171.81) | 119.42  (73.76, 182.02) | 91.52  (55.91, 143.08) | 101.16  (62.77, 158.27) | 131.74  (80.36, 201.3) | 134.9  (84.27, 206.12) |
| Other mental disorders | 96.49  (60.83, 147.36) | 106.04  (67.06, 161.37) | 111.75  (69.46, 170.82) | 127.5  (80.24, 194.79) | 82.59  (51.57, 126.36) | 87.96  (55.09, 133.03) |
| Attention-deficit  hyperactivity disorder | 8.65  (4.81, 14.81) | 7.68  (4.26, 13.15) | 12.83  (6.93, 22.26) | 11.18  (6.07, 19.1) | 4.84  (2.56, 8.35) | 4.38  (2.32, 7.55) |
| Conduct disorder | 69.9  (38.06, 111.17) | 58.9  (32, 93.8) | 98.11  (54.23, 153.31) | 78.13  (43.3, 122.24) | 44.21  (22.59, 73.52) | 39.35  (20.21, 65.98) |
| Bipolar disorder | 80.45  (46.88, 124.08) | 82.26  (48.93, 126.33) | 82.32  (47.88, 128.47) | 86.83  (51.15, 136.09) | 78.74  (45.83, 121.3) | 78.42  (46.46, 122.16) |
| Autism spectrum disorders | 45.11  (29.51, 66.35) | 43.9  (28.65, 64.13) | 66.15  (42.58, 97.14) | 64.12  (41.51, 94.04) | 25.94  (16.5, 37.88) | 25.39  (16.08, 37.01) |
| Schizophrenia | 159.38  (110.72, 212.83) | 169.46  (118.16, 224.01) | 174.32  (121.56, 232.96) | 194.55  (135.54, 258.39) | 145.78  (100.72, 197.67) | 148.73  (103.92, 199.7) |
| Bulimia nervosa | 16.81  (9.54, 27.04) | 15.34  (8.67, 24.4) | 13.14  (7.26, 21.37) | 12.53  (6.91, 20.21) | 20.15  (11.57, 32.09) | 17.67  (10, 28.15) |
| Anorexia nervosa | 7.76  (4.35, 13.02) | 6.71  (3.77, 11.18) | 3.99  (2.03, 7.02) | 3.46  (1.79, 5.97) | 11.19  (6.09, 18.74) | 9.53  (5.22, 15.87) |
