# Supplementary material for: The burden of mental disorders in Nepal between 1990 and 2019: Findings from the Global Burden of Disease Study 2019
Source: Glob Ment Health (Camb). 2023 Sep 12;10:e61. doi: 10.1017/gmh.2023.55 (PMC10579670; doi:10.1017/gmh.2023.55)
Supplement: Dhungana et al. supplementary material 2 — Dhungana et al. supplementary material [file S2054425123000559sup002.docx]

**Supplementary file 2. DALYs lost due to mental disorders between 1990 and 2019**

|  | **Both sex** | | | **Male** | | | **Female** | | |
| --- | --- | --- | --- | --- | --- | --- | --- | --- | --- |
|  | **All ages** | **age standardized** | **% of total DALYS** | **All ages** | **age standardized** | **% of total DALYS** | **All ages** | **age standardized** | **% of total DALYS** |
| **1990** | 1421.66  (1036.74, 1870.04) | 1739.45  (1285.35, 2287.93) | 1.79  (1.34, 2.32) | 1375.63  (997.36, 1803.81) | 1665.07  (1232.96, 2171.55) | 1.7  (1.26, 2.21) | 1467.92  (1068.11, 1944.53) | 1814.42  (1329.34, 2396.07) | 1.89  (1.41, 2.44) |
| **1995** | 1414.42  (1036.32, 1861.19) | 1720.37  (1278.89, 2261.04) | 2.24  (1.65, 2.89) | 1370.19  (990.41, 1806.12) | 1649.38  (1211.89, 2173.98) | 2.12  (1.56, 2.74) | 1458.8  (1063.45, 1929.83) | 1791.28  (1320.04, 2350.7) | 2.38  (1.76, 3.04) |
| **2000** | 1502.69  (1103.09, 1980.14) | 1807.28  (1344.53, 2375.17) | 3.07  (2.32, 3.97) | 1430.49  (1039.03, 1871.39) | 1700.54  (1248.64, 2221.07) | 2.81  (2.1, 3.63) | 1575.08  (1158.01, 2083.93) | 1914.34  (1415, 2534.02) | 3.36  (2.55, 4.32) |
| **2005** | 1556.85  (1140.29, 2056.8) | 1816.41  (1342.39, 2391.03) | 3.9  (2.96, 4.92) | 1461.88  (1074.97, 1920.99) | 1694.14  (1239.53, 2217.52) | 3.47  (2.63, 4.44) | 1650.06  (1203.7, 2166.8) | 1935.43  (1417.36, 2542.37) | 4.36  (3.32, 5.48) |
| **2010** | 1636.35  (1203.26, 2147.28) | 1846.03  (1370.45, 2417.45) | 4.68  (3.62, 5.9) | 1512.26  (1115.56, 1995.31) | 1709.91  (1260.47, 2253.64) | 4.08  (3.13, 5.17) | 1754.37  (1288.29, 2298.86) | 1971.05  (1450.49, 2588.47) | 5.33  (4.1, 6.68) |
| **2015** | 1636.44  (1199.32, 2160.23) | 1766.57  (1310.42, 2337.09) | 4.83  (3.7, 6.1) | 1510.85  (1102.57, 2007.79) | 1652.74  (1208.28, 2177.99) | 4.12  (3.14, 5.25) | 1752.71  (1284.28, 2316.69) | 1864.48  (1373.33, 2471.36) | 5.61  (4.32, 7.09) |
| **2019** | 1691.08  (1244.47, 2224.51) | 1773.74  (1309.23, 2335.08) | 5.53  (4.22, 6.98) | 1545.37  (1129.01, 2027.12) | 1653.62  (1210.22, 2171.39) | 4.73  (3.58, 6) | 1823.78  (1330.85, 2413) | 1873.84  (1382.59, 2478.2) | 6.37  (4.89, 7.96) |
