# Supplementary material for: The burden of mental disorders in Nepal between 1990 and 2019: Findings from the Global Burden of Disease Study 2019
Source: Glob Ment Health (Camb). 2023 Sep 12;10:e61. doi: 10.1017/gmh.2023.55 (PMC10579670; doi:10.1017/gmh.2023.55)
Supplement: Dhungana et al. supplementary material 3 — Dhungana et al. supplementary material [file S2054425123000559sup003.docx]

Table 1. DALYs attributable to specific mental disorders (100,000 population)

|  | **Both sex** | | **Male** | | **Female** | |
| --- | --- | --- | --- | --- | --- | --- |
|  | **All ages** | **Age standardized** | **All ages** | **Age standardized** | **All ages** | **Age standardized** |
| Major depressive disorder | 687.86  (465.55, 953.1) | 754.62  (510.96, 1045.8) | 531.59  (350.9, 739.48) | 605.29  (403.62, 842.67) | 830.19  (560.27, 1159.32) | 883.21  (598.08, 1228.91) |
| Anxiety disorders | 302.23  (199.03, 429.32) | 309.51  (205.76, 434.63) | 245.44  (162.14, 345.61) | 259.69  (172.72, 364.95) | 353.95  (230.59, 507.35) | 352.97  (229.71, 498.14) |
| Idiopathic developmental  intellectual disability | 103.87  (57.01, 168.63) | 99.9  (54.97, 162.85) | 114.21  (62.07, 186.85) | 109.17  (59.37, 178.21) | 94.46  (52.36, 152.89) | 91.33  (50.67, 147.78) |
| Dysthymia | 112.57  (68.7, 171.81) | 119.42  (73.76, 182.02) | 91.52  (55.91, 143.08) | 101.16  (62.77, 158.27) | 131.74  (80.36, 201.3) | 134.9  (84.27, 206.12) |
| Other mental disorders | 96.49  (60.83, 147.36) | 106.04  (67.06, 161.37) | 111.75  (69.46, 170.82) | 127.5  (80.24, 194.79) | 82.59  (51.57, 126.36) | 87.96  (55.09, 133.03) |
| Attention-deficit  hyperactivity disorder | 8.65  (4.81, 14.81) | 7.68  (4.26, 13.15) | 12.83  (6.93, 22.26) | 11.18  (6.07, 19.1) | 4.84  (2.56, 8.35) | 4.38  (2.32, 7.55) |
| Conduct disorder | 69.9  (38.06, 111.17) | 58.9  (32, 93.8) | 98.11  (54.23, 153.31) | 78.13  (43.3, 122.24) | 44.21  (22.59, 73.52) | 39.35  (20.21, 65.98) |
| Bipolar disorder | 80.45  (46.88, 124.08) | 82.26  (48.93, 126.33) | 82.32  (47.88, 128.47) | 86.83  (51.15, 136.09) | 78.74  (45.83, 121.3) | 78.42  (46.46, 122.16) |
| Autism spectrum disorders | 45.11  (29.51, 66.35) | 43.9  (28.65, 64.13) | 66.15  (42.58, 97.14) | 64.12  (41.51, 94.04) | 25.94  (16.5, 37.88) | 25.39  (16.08, 37.01) |
| Schizophrenia | 159.38  (110.72, 212.83) | 169.46  (118.16, 224.01) | 174.32  (121.56, 232.96) | 194.55  (135.54, 258.39) | 145.78  (100.72, 197.67) | 148.73  (103.92, 199.7) |
| Bulimia nervosa | 16.81  (9.54, 27.04) | 15.34  (8.67, 24.4) | 13.14  (7.26, 21.37) | 12.53  (6.91, 20.21) | 20.15  (11.57, 32.09) | 17.67  (10, 28.15) |
| Anorexia nervosa | 7.76  (4.35, 13.02) | 6.71  (3.77, 11.18) | 3.99  (2.03, 7.02) | 3.46  (1.79, 5.97) | 11.19  (6.09, 18.74) | 9.53  (5.22, 15.87) |
